# Hypoxia inducible factor-1α correlates with vascular endothelial growth factor A and C indicating worse prognosis in clear cell renal cell carcinoma

**DOI:** 10.1186/1756-9966-28-40

**Published:** 2009-03-20

**Authors:** Gordana Đorđević, Koviljka Matušan-Ilijaš, Emina Babarović, Ita Hadžisejdić, Maja Grahovac, Blaženka Grahovac, Nives Jonjić

**Affiliations:** 1 Department of Pathology, Rijeka University School of Medicine, Braæe Branchetta 20, HR-51000 Rijeka, Croatia

## Abstract

**Background:**

The role of angiogenesis in the pathogenesis of renal cell carcinoma is well recognized, however, the influence of tumor cells in this activity has not yet been fully clarified. The aim of this study was to analyze the expression of hypoxia inducible factor-1α (HIF-1α), a regulatory factor of angiogenic switch, in comparison to vascular endothelial growth factor A and C (VEGF-A and VEGF-C), recognized to be involved in blood and lymph vessel neoangiogenesis, with potential association in the prognosis of patients with renal cell carcinoma.

**Methods:**

Ninety-four patients with diagnosis of clear cell renal cell carcinomas (CCRCC), all clinicopathological characteristics and overall survival were unrolled in this study. Immunohistochemicaly VEGF-A, VEGF-C, HIF-1α and Ki67 were detected on tumor cells and the staining was performed on tissue microarrays (TMA). The staining was evaluated as a percentage of cytoplasmic or nuclear positive tumor cells.

**Results:**

Variable expression of all three proteins was confirmed. Both angiogenic factors demonstrated perimembranous or diffuse cytoplasmic staining, with diffuse pattern positively associated (p < 0.001). Nuclear HIF-1α expression (nHIF-1α) showed inverse correlation with diffuse cytoplasmic VEGF-A (p = 0.002) and VEGF-C (p = 0.053), while cytoplasmic HIF-1α expression (cHIF-1α) showed positive correlation with diffuse staining of both angiogenic factors (p < 0.001; p < 0.001, respectively). In comparison to clinicopathological characteristics, a higher nuclear grade (p = 0.006; p < 0.001, respectively), larger tumor size (p = 0.009; p = 0.015, respectively), higher stage (p = 0.023; p = 0.027, respectively) and shorter survival (p = 0.018; p = 0.024, respectively) were associated with overexpression of cHIF-1α and diffuse cytoplasmic VEGF-A expression. In contrary, overexpression of nHIF-1α was associated with better diagnostic parameters i.e. lower nuclear grade (p = 0.006), smaller tumor size (p = 0.057), and longer survival (p = 0.005).

**Conclusion:**

Overexpression of VEGF-A and cHIF-1α in tumor cells highlights a more aggressive subtype of CCRCC that might have some clinical implications. The significance of nHIF-1α expression associated with better differentiated tumors should be further elucidated.

## Background

Angiogenesis plays an important role in the development, progression and dissemination of human tumors [1]. In the last decade, many angiogenic factors and their receptors have been shown to be expressed in renal cell carcinoma (RCC) [2]. Among three dominating types of RCC, clear cell RCC (CCRCC) is generally more vascularized than the papillary and chromophobe types [3,4]. This vascularization is most likely due to the biallelic loss of the von Hippel Lindau (VHL) tumor suppressor gene which is associated with 50–80% of sporadic CCRCC [5,6]. It is clear that VHL gene encodes the pVHL, a component of E3 ubiquitin ligase, important in the ubiquitin-proteasome protein degradation mechanism that targets hypoxia inducible factors HIF-1α and HIF-2α [7].

HIF-1α is a heterodimeric transcription factor, and its products regulate cell adaptation to hypoxic stress by modulating a number of genes involved in vascular growth and cellular metabolism, such as vascular endothelial growth factors (VEGFs), erythropoietin or glucose transporter-1 in physiologic and pathologic conditions [8,9]. VEGFs include distinct signaling pathways for angiogenesis and lymphangiogenesis and structurally belong to the platelet derived growth factor family (PDGF). Several closely related proteins have been discovered (VEGF A-F) [1]. VEGF, sometimes referred to as VEGF-A, has been shown to stimulate endothelial cell mitogenesis and cell migration as well as vasodilatation and vascular permeability [10].

VEGF-C is an essential chemotactic and survival factor during embryonic and inflammatory lymphangiogenesis and is predominantly expressed along with the VEGFR-3 receptor. There is evidence that tumor cells and tumor associated macrophages secrete lymphangiogenic growth factor VEGF-C, which induces development of nearby lymphatic vessels, facilitating the access of tumor cells into the vessels [11]. VEGF-C mRNA has been detected in adult human kidney where it acts in an autocrine manner to promote survival in podocytes [12], and is one of the potential regulators of proximal tubular epithelial cell communication with the peritubular capillary network [13,14]. Literature data on the expression of VEGF-C in CCRCC are controversial, mostly suggesting that VEGF-C plays a little role in the progression of RCC [2].

Our previous studies demonstrated a heterogeneous expression of VEGF-A in CCRCC with two distinct staining patterns being associated with different clinicopathologic characteristics [15]. The aim of this study was to expand our knowledge on the expression of VEGF-C, recognized to be involved in lymph vessel neoangiogenesis, and to compare its value with the VEGF-A expression. Furthermore, the expression of both angiogenic factors was analyzed in comparison to HIF-1α, a regulatory factor of angiogenic switch, and finally all study parameters were compared with clinicopathologic characteristics of CCRCC including patient survival.

## Methods

### Clinicopathologic data

This study included tumor specimens of CCRCC obtained from patients undergoing nephrectomy at Department of Urology, Rijeka University Hospital Center in Rijeka. All cases were reviewed by two pathologists using WHO tumor classification criteria [3]. Tissue microarrays (TMA) were built from 94 archive formalin fixed and paraffin embedded tumor tissues collected consecutively from 1989 to 1994. Clinicopathologic data obtained from patient medical records and from files kept at Department of Pathology, Rijeka University School of Medicine, Rijeka, Croatia, included sex, age, overall survival, tumor size, TNM stage, histological subtype and nuclear grade as assessed using Fuhrman nuclear grading system [16].

### Tissue microarray (TMA) construction

Hematoxylin and eosin stained tumor sections were used to mark areas with highest nuclear grade avoiding areas of necrosis. For all cases two donor blocks of each carcinoma were used. Three tissue cores, each 1 mm in diameter, were placed into recipient paraffin block using a manual tissue arrayer (Alphelys, Plaisir, France). Normal liver tissue was used for orientation. Cores were spaced at intervals of 0.5 mm in the x- and y-axes. One section from each TMA block was stained with hematoxylin and eosin for morphological assessment. Serial sections were cut from TMA blocks for immunhistochemical staining. Five-μm thick sections were placed on adhesive glass slides (Capillary Gap Microscope Slides, 75 μm, Code S2024, DakoCytomation, Glostrup, Denmark), left to dry at 37°C overnight and stored in the dark at +4°C.

### Immunohistochemistry

Tumor samples were processed for immunohistology analysis in a Dako Autostainer Plus (DakoCytomation Colorado Inc, Fort Collins, CO, USA) according to the manufacturer's protocol using Envision peroxidase procedure (ChemMate TM Envision HRP detection kit K5007, DakoCytomation, Glostrup, Denmark). Epitope retrieval for VEGF-A, VEGF-C and Ki67 was achieved by immersing slides in Tris-EDTA buffer (pH 9.0) and boiling for 10 minutes in water bath and for HIF-1α by immersing slides in citrate buffer (pH 6.0) and boiling for 45 minutes. The slides were allowed to cool for 45 minutes and then preincubated with blocking solution containing normal goat serum (DakoCytomation, Glostrup, Denmark) for 30 minutes. Primary antibodies, anti-HIF-1α (NB 100–131, Novus Biologicals, Littleton, CO, USA, dilution 1:3000, 30 min incubation), and anti-VEGF-A (C-1: sc-7269, Santa Cruz Biotechnology, Santa Cruz, CA, USA, dilution 1:500, overnight incubation at 4°C) were monoclonal antibodies of mouse origin, while anti-VEGF-C (H-190: sc-9047, Santa Cruz Biotechnology, Santa Cruz, CA, USA, dilution 1:100, overnight incubation at 4°C) was polyclonal antibody of rabbit origin. Proliferative activity was evaluated by detecting the Ki67 protein with monoclonal antibody (clone MIB-1, DakoCytomation, Glostrup, Denmark, dilution 1:50, 30-min incubation). The binding of the primary antibodies was assessed by incubation of secondary antibody (Dako REAL EnVision™/HRP, Rabbit/Mouse (ENV) K5007, DakoCytomation, Glostrup, Denmark, 30-min incubation). A negative control consisting of the omission of the primary antibody was performed for each case.

### Evaluation of immunostaining

The immunohistochemical staining results were evaluated independently by two pathologists, without knowledge of clinicopathologic data on each individual case. No interobserver variability was found between the results of the two independent observers. On statistical analysis, the mean value of immunohistochemical staining of all three tissue microarrays was used.

HIF-1α immunoreactivity was evaluated as percentage of nuclear or cytoplasmic positivity by counting positive tumor nuclei/cytoplasm at 500 tumor cells in tumor areas with highest density of positive cells using ×400 magnification and ISSA 3.1 software (Vams, Zagreb, Croatia). The immunostaining of VEGF-A and C was evaluated as percentage of diffuse and perimembranous cytoplasmic staining pattern in tumor cells. Smooth muscle cells in vascular walls were used as internal control for VEGF-A, cortical tubular cells for VEGF-C and glioblastoma cells that were usually intensively positive when palisading around necroses for HIF-1α. Ki67 index was also quantified by ISSA 3.1 software (Vams, Zagreb, Croatia) and assessed by scoring 500 tumor cells at ×400 magnification in the region with highest proliferative activity.

### Statistical analysis

Statistical analysis was performed using Statistica 6.1 software (StatSoft, Inc., Tulsa, OK, USA). Mann-Whitney U-test was used to assess the significance of association of HIF-1α, VEGF-A and -C with clinicopathologic data such as nuclear grade, tumor size, Ki67 index and pathologic stage. Pearson's correlation was used to determine association between HIF-1α and VEGF-A or -C. The association of immunohistochemical staining for HIF-1α, VEGF-A and -C with patient survival was evaluated using Kaplan-Meier method, and differences between groups were tested by the log-rank test. Statistical differences with p value less than 0.05 were considered significant.

## Results

### Immunoreacitivty of HIF-1α, VEGF-A and -C in clear cell renal cell carcinoma

#### HIF-1α

**Figure 1:**
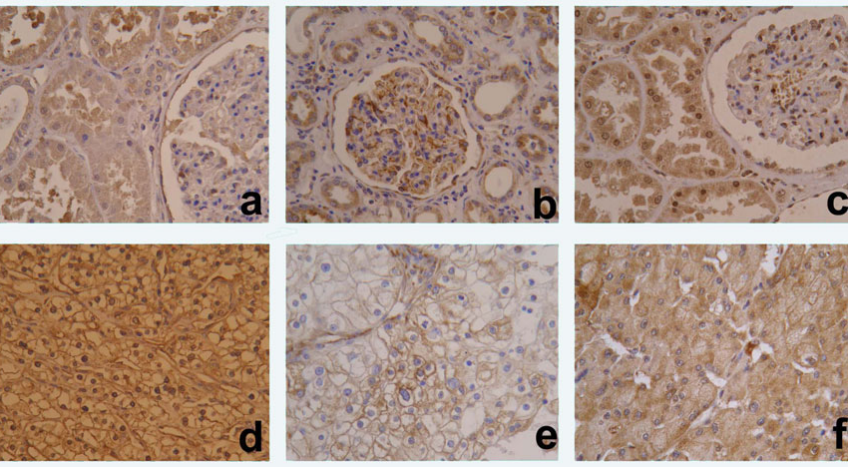
**Immunohistochemical staining of HIF-1α, VEGF-A and VEGF-C in normal renal tissue (A-C) and clear cell renal cell carcinoma (CCRCC) (D-F)**. A homogeneous cytoplasmic staining of tubular cells and weak staining in glomerules was observed with HIF-1α (A), while VEGF-A and VEGF-C were positive in tubular cells, glomerular mesangium and interstitial macrophages (B and C). In CCRCC, HIF-1α immmunoreactivity was nuclear and/or cytoplasmic (D), while it was perimembranous and/or diffuse cytoplasmic for VEGF-A and VEFG-C (E and F). (magnification ×200).

In normal renal tissue, there was diffuse cytoplasmic staining of tubular cells and weak, nonspecific immunostaining in mesangial area in some glomeruli, which we claimed as being negative for HIF-1α. In CCRCC, staining was present in both tumor cell nuclei and/or cytoplasm ranging from low to strong intensity (Fig. 1). Tumors showed different proportions of positive nuclei (nHIF-1α) and cytoplasm (cHIF-1α) for HIF-1α antibody (median value 47.1, range 16.3–82.3 and median value 12.9, range 1.4–75, respectively).

#### VEGF-A and C

Immunohistochemical staining of VEGF-A was cytoplasmic, both in normal renal tissue and tumor cells, as we described previously [15]. Immunohistochemical staining of VEGF-C was also cytoplasmic in normal renal tissue and CCRCC showing heterogeneous staining of different intensity and percentage of positive tumor cytoplasm as well as perimembranous and/or diffuse staining pattern (Fig. 1). Division according to percentage of perimembranous or diffuse staining pattern turned out to be more important than intensity and/or percentage of positive tumor cytoplasm in relation to HIF-1α or clinicopathologic parameters. The median value of perimembranous staining pattern was 12.7% (range 0–94%) for VEGF-A (pVEGF-A) and 46% (range 0–100%) for VEGF-C (pVEGF-C). The median value of diffuse cytoplasmic pattern was 10% (range 0–92%) for VEGF-A (dVEGF-A) and 26.3% (range 0–100%) for VEGF-C (dVEGF-C).

### Association between HIF-1α, VEGF-A and -C

Nuclear HIF-1α demonstrated inverse correlation with dVEGF-A (p = 0.002) and almost so with dVEGF-C (p = 0.053), and showed no association with perimembranous staining pattern of either VEGF-A or -C. Cytoplasmic HIF-1α correlated with both dVEGF-A (p < 0.001) and dVEGF-C (p = <0.001), and also showed inverse correlation with perimembranous staining pattern of VEGF-C (p < 0.001), but not VEGF-A (Table 1).

**Table 1:** Relation of HIF-1α to VEGF-A and VEGF-C

		VEGF-A (%)				VEGF-C (%)			
		pVEGF-A		dVEGF-A		pVEGF-C		dVEGF-C	
		
		p^1^	r_p_^1^	p^1^	r_p_^1^	p^1^	r_p_^1^	p^1^	r_p_^1^
HIF-1α (%)	nHIF-1α	0.535	0.068	0.002	-0.322	0.121	0.168	0.053	-0.209
	cHIF-1α	0.094	-0.180	<0.001	0.526	<0.001	-0.629	<0.001	0.637

^1^Pearson's correlation

Regarding association of VEGF-A and -C, Pearson's correlation showed a relation of only diffuse staining pattern of both proteins (p < 0.001, r_p _= 0.586) with no association between the perimembranous staining patterns of the mentioned growth factors.

### Association of HIF-1α, VEGF-A and -C with clinicopathologic parameters

There were 59 men and 35 women in the study. The median value of tumor size was 6.3 (1.8–17.5) cm. The Fuhrman nuclear grading distribution was as follows: 12 (12.8%) grade 1, 40 (42.6%) grade 2, 22 (23.4%) grade 3 and 20 (21.2%) grade 4 tumors. There were 71 (75.5%) tumors limited to the kidney (pT1 and pT2) and 23 (24.5%) tumors with extrarenal expansion (pT3 and pT4). The mean value of Ki67 proliferative index was 10.7 ± 5.9%. Follow up was available for 87 patients and ranged from 1 to 165 months (median 64 months). Survival time was calculated from the date of surgery to the date of death or of the last follow up.

The expression of HIF-1α, VEGF-A and VEGF-C in carcinoma cells was compared to tumor variables that represent prognostic factors in CRCC: nuclear grade, tumor size, Ki67 proliferative index and pathologic stage (Table 2).

**Table 2:** Relation of HIF-1α, VEGF-A and VEGF-C to clinicopathologic parameters

		Nuclear grade^1^		*P *value	Tumor size (cm)^1^		p value	Ki67 (%)^1,2^		*P *value	Pathologic stage^1^		*P *value
		1,2	3,4		< 7	≥ 7		low	high		1	2,3,4,	
HIF-1α	nHIF-1α	49.5	39	0.006	48.6	43.6	0.057	43.9	48.1	0.134	48.1	44.5	0.165
(%)		(16.3–82.3)	(19.2–72.6)		(27.9–73.9)	(16.3–82.3)		(16.3–72.4)	(21.2–82.3)		(27.9–73.9)	(16.3–82.3)	

	cHIF-1α	11.4	18.7	0.006	11.3	17.5	0.009	14.6	11.6	0.246	11.4	16.6	0.023
		(1.4–75)	(5.2–59.5)		(1.4–59.5)	(2.9–75)		(4.3–75)	(1.4–46.5)		(1.4–42.6)	(2.9–75)	

VEGF-A	pVEGF-A	15	12.5	0.307	15	7.5	0.173	12.5	12.7	0.658	12.1	17.5	0.682
(%)		(0.00–94)	(0–75)		(0–94)	(0–75)		(0–94)	(0–75)		(0–94)	(0–75)	

	dVEGF-A	6.7	30	<0.001	6.7	16.7	0.015	10.6	10	0.652	6.3	11.7	0.027
		(0–92.5)	(0–90)		(0–67.5)	(0–92.5)		(0–92.5)	(0–83.3)		(0–76.7)	(0–92.5)	

VEGF-C	pVEGF-C	65	14	<0.001	64.2	27.9	0.007	45	55	0.913	61.3	33.3	0.042
(%)		(0–100)	(0–92.5)		(0–100)	(0–100)		(0–100)	(0–100)		(0–100)	(0–100)	

	dVEGF-C	18.5	37	0.004	18	37.1	0.007	25	26.3	0.516	20	30	0.109
		(0–100)	(0–100)		(0–100)	(0–100)		(0–100)	(0–100)		(0–100)	(0–100)	

^1^Mann-Whitney U-test; median (range); ^2^cut-off is mean

Nuclear HIF-1α and pVEGF-C expression was associated with lower nuclear grade and smaller tumor size indicating better prognosis, while cHIF-1α together with dVEGF-A and -C was associated with worse prognostic factors, i.e. higher nuclear grade, larger tumor size and higher tumor stage. There was no association of Ki67 index with either protein analyzed.

### Association of HIF-1α, VEGF-A and -C with patient survival

**Figure 2:**
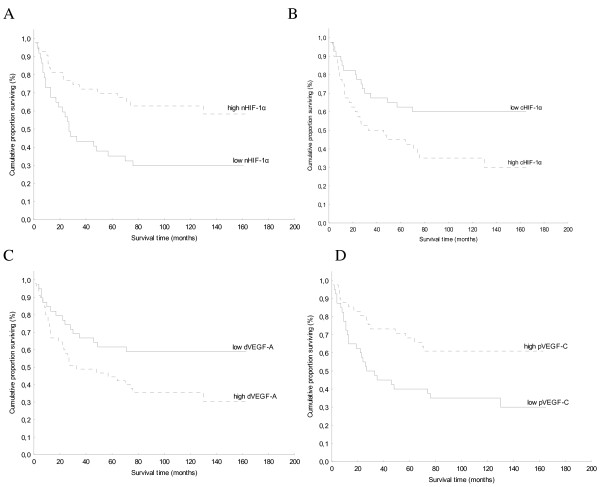
**Kaplan-Meier cumulative survival analysis according to staining for nuclear and cytoplasmic HIF-1α, VEGF-A and VEGF-C**. The log-rank test showed significantly shorter overall survival in patients with tumors showing low nHIF-1α (p = 0.005) (A) and low pVEGF-C (p = 0.008) (D). The 5-year survival rate was 32% for patients whose tumors showed low nHIF-1α *vs*. 65% for patients whose tumors showed high nHIF-1α (A); and 40% for patients whose tumors showed low pVEGF-C *vs*. 61% for patients whose tumors showed high pVEGF-C (D). The log-rank test showed significantly shorter overall survival in patients with tumors showing high cHIF-1α (p = 0.018) (B) and high dVEGF-A (p = 0.024) (C). The 5-year survival rate was 60% for patients whose tumors showed low cHIF-1α *vs*. 40% for patients whose tumors showed high cHIF-1α (B); and 59% for patients whose tumors showed low dVEGF-A *vs*. 40% for patients whose tumors showed high dVEGF-A (C).

The association of immunohistochemical positivity for HIF-1α, VEGF-A and VEGF-C and cumulative proportion of patients surviving during the follow up are shown in Figure 2.

The 5-year survival rates were significantly shorter for patients whose tumors demonstrated low percentage of nHIF-1α and pVEGF-C and high percentage of cHIF-1α and dVEGF-A. Because tumor grading and staging are considered as major prognostic parameters in CCRCC, we first analyzed their impact on postoperative survival. We found a significant inverse association between survival and tumor grading (p < 0.001) or staging (p = 0.003). Univariate survival analysis showed nuclear grade, pathologic stage, nHIF-1α and cHIF-1α expression as well as pVEGF-C and dVEGF-A to be significant predictive factors. However, on multivariate analysis only nuclear grade remained significant (relative risk was 3 and 95% confidence interval 1.7–5.3), while pathologic stage (relative risk was 1.5 and 95% confidence interval 1–2.4) together with immunohistochemically analyzed proteins showed no independent prognostic value.

## Discussion

There is a very large body of evidence that VEGF-A and related molecules such as VEGF-C and VEGF-D are potent proangiogenic factors involved in tumor growth and metastasis. Their intra-cell signaling pathway through specific receptors (VEGFRs) with tyrosine kinase activity provides targets for novel antiangiogenic designed drugs [10,11,17]. Our study demonstrated the expression of VEGF-A and VEGF-C on tumor cells but also in the cytoplasm of cortical tubular cells, endothelium, mesangium and macrophages, which is consistent with literature reports [12-14,18]. Endothelial-cell maintenance through regulated VEGF levels is crucial for glomerular function [19]. VEGF-C promotes survival in podocytes acting in an autocrine manner and both factors probably coordinate the synchronous development of the tubular and vascular architecture in the kidney required for the formation of the functioning nephron [12-14].

Similar to our previous work [15] on whole tumor slices, the heterogeneous expression of VEGF-A was also confirmed in TMA technique. Both angiogenic cytokines were immunohistochemically detected as heterogeneous staining of different intensity and percentage of positive tumor cells. Attention was especially focused on the pattern of their cytoplasmic distribution, diffuse and/or perimembranous, as previously reported by Yildis *et al*. [20] and Jacobsen *et al*. [21]. Jacobsen *et al*. believed that immunohistochemical VEGF expression near the cell membrane was affected by storage time of paraffin embedded tumor specimens and this type of VEGF expression was not further evaluated [21]. According to our results, the percentage of perimembranous or diffuse staining pattern turned out to be more important than the intensity and/or percentage of positive tumor cells in relation to HIF-1α or pathological and clinical parameters relevant for disease prognosis. Namely, diffuse and intensive cytoplasmic VEGF-A and -C staining was associated with higher nuclear grade, larger tumor size, higher tumor stage and higher cHIF-1α.

There are not so many reports on VEGF-C expression in CCRCC. Gunningham *et al*. found no significant up-regulation of VEGF-C in neoplastic tissue compared with normal kidney [2]. According to Leppert *et al*., there was no difference in the expression of VEGF-C among three main types of RCC, although its main receptor VEGF-R3 was overexpressed in CCRCC [22]. Also, a reduction of mRNA VEGF-C in tumors was observed; however, it was not biologically significant [2]. Recent results reported by Iwata *et al*. [10] showed no significant relationship between VEGF-C expression and clinicopathologic features of RCC, while we found diffuse cytoplasmic and perimembranous distribution to be associated with different clinicopathologic parameters. Moreover, survival analysis showed a significantly shorter overall survival in patients with tumors exhibiting high diffuse cytoplasmic staining of VEGF-A/C. This controversial but statistically consistent result may suggest that detection of the cytoplasmic pattern in immunohistochemical distribution of VEGF-C could possible mean activation of various mechanisms in the progression of CCRCC.

Regarding HIF-1α expression in normal renal parenchyma, there was no positive reaction in glomeruli and no nuclear positivity in normal tubular epithelium, as reported by Di Cristofano *et al*. [23]. In CCRCC, the expression was nuclear and/or cytoplasmic ranging from low to strong intensity. Some authors report on protein expression of HIF-1α in the tissue of RCC to be significantly higher than in renal parenchyma adjacent to the cancer [24]. The present study demonstrated correlation of overexpression of all three proteins analyzed, i.e. HIF-1α, VEGF-A and VEGF-C. Both nuclear and diffuse cytoplasmic positivity was statistically important in comparison with angiogenic factor expression and clinicopathologic parameters. Nuclear HIF-1α expression was associated with better prognosis in CCRCC, while cHIF-1α was related to worse prognostic factors and shorter patient survival. Recent literature data on the expression of this regulatory factor are still controversial. According to Kubis *et al*., up-regulation of the angiogenic genes is due to an increase of HIF-1α protein levels in the cytoplasm by inhibition of its targeting for proteosomal degradation and not by regulation of nuclear import by its nuclear location signal [25]. Lindgren *et al*. did not evaluate nuclear staining and found the cHIF-1α levels in patients with CCRCC to be significantly lower in locally aggressive tumors than in localized tumors [26]. Klatte *et al*. conclude that high nHIF-1α expression significantly correlates with markers of apoptosis, VEGFs, and worse survival as compared with patients with low nuclear expression, which was demonstrated by multivariate analysis [24]. Di Cristofano *et al*. noted that in VHL inactivated tumors, strong cytoplasmic positivity implied favorable prognosis, while strong nuclear localization of HIF-1α was associated with worse tumor specific survival [23].

## Conclusion

Our results on nuclear expression of HIF-1α were quite opposite to studies that describe nHIF-1α overexpression as a marker of unfavorable prognosis in human cancer [27-29]. Discrepancies between studies may reflect the balance of multiple effects of HIF status with compartmentalization according to specific functional moments. The HIF-1α mediated hypoxia response is therefore complex and different pathways are likely to be activated in different cell types.

In conclusion, the results obtained in this study highlight the more aggressive subtype of CCRCC, associated with overexpression of VEGF-A and cHIF-1α, which may have some clinical implication. Additional studies are needed to understand the significance of nHIF-1α expression associated with better-differentiated tumors.

## Competing interests

The authors declare that they have no competing interests.

## Authors' contributions

GĐ conceived of the study and drafted the manuscript. KMI participated in the design of the study, carried out the immunoassays and performed the statistical analysis. EB carried out the immunoassays, participated in the sequence alignment and helped to draft the manuscript. IH, MG and BG carried out the molecular studies and participated in the sequence alignment. NJ conceived of the study, and participated in its design and coordination. All authors read and approved the final manuscript.
